# Overexpression of IL-15 promotes tumor destruction via NK1.1+ cells in a spontaneous breast cancer model

**DOI:** 10.1186/s12885-015-1264-3

**Published:** 2015-04-16

**Authors:** Amy E Gillgrass, Marianne V Chew, Tamara Krneta, Ali A Ashkar

**Affiliations:** Department of Pathology and Molecular Medicine, McMaster Immunology Research Center (MIRC), McMaster University, Hamilton, Canada

**Keywords:** NK cells, Breast cancer, IL-15, CD8 T cells, NK1.1

## Abstract

**Background:**

Natural Killer (NK) cells play an important role in tumor prevention, but once tumors form, the numbers as well as the cytotoxic functions of NK cells are reduced. IL-15 is a cytokine that increases and activates NK cells. Here we will examine the anti-tumor role of IL-15 in a spontaneous breast cancer model.

**Methods:**

To achieve this, Polyoma Middle T (MT) mice that form spontaneous breast cancer were crossed with mice that either overexpress IL-15 (IL-15 transgenic (TG)) or mice that lack IL-15 (IL-15 knockout (KO)). We compared survival curves and tumor formation in IL-15 KO/MT, MT and IL-15 TG/MT groups. In addition, the phenotype, activation and contribution of NK cells and CD8 T cells to tumor formation were examined in each of these mouse strains via flow cytometry, ELISA, adoptive transfer and antibody depletion experiments.

**Results:**

IL-15KO/MT tumors formed and progressed to endpoint more quickly than MT tumors. These tumors displayed little apoptosis and poor CD8 T cell infiltration. In contrast, IL-15 TG/MT mice had increased survival and the tumors displayed extensive cell death, high proportions of activated NK cells and a higher infiltration of CD8 T cells than MT tumors. CD8 T cells in IL-15 TG/MT tumors were capable of secreting IFNγ, possessed markers of memory, did not display an exhausted phenotype and were frequently NK1.1+. Long-term antibody depletion studies in IL-15 TG/MT mice revealed that NK1.1+, but not CD8 T cells, were critical for tumor destruction. Lastly, human NK cells, when exposed to a similar cytokine environment as that found in IL-15TG/MT tumors, were capable of killing human breast cancer cells.

**Conclusions:**

This study reveals that high levels of IL-15 can promote tumor destruction and reduce metastasis in breast cancer via effects on NK1.1+ cells. Our results suggest that strategies aimed at increasing NK cell activation may be effective against solid epithelial cancers.

**Electronic supplementary material:**

The online version of this article (doi:10.1186/s12885-015-1264-3) contains supplementary material, which is available to authorized users.

## Background

Natural Killer (NK) cells were discovered almost 40 years ago due to their ability to kill tumor cells with no prior sensitization [1]. Since then, extensive knowledge has been gained about their instrumental role in tumor immunosurveillance [2]. NK cells are capable of killing tumor cells via multiple mechanisms. The ability of an NK cell to kill another cell is controlled by a balance of activating and inhibitory receptors expressed on their cell surface that allow the NK cells to sense self versus damaged cells [3]. Recently, it has been shown that in addition to preventing tumor formation, NK cells can eradicate large solid tumors [4] and kill mammary cancer stem cells [5]. Unfortunately, in several malignancies, including breast cancer, NK cell activity as well as expression of activating receptors, is often suppressed [6,7]. Tumors promote this down regulation by the secretion of molecules such as TGF β and IL-10 [7-9]. Recent reports suggest that NK cells within the tumor may actually support tumor growth [10,11]. Alterations in NK cell activity are reversible, as NK cells rapidly respond to their environment [7,12]. The ability to shift the NK cell phenotype from inhibition/tumor promotion to activation will be essential for the use of NK cells against cancer.

IL-15 is a cytokine that has effects on both the innate and the adaptive immune system. IL-15 promotes the differentiation, proliferation and activation of NK cells and the formation of a subset of memory CD8 T cells [13-15]. This has been confirmed in IL-15 TG mice which have increased activated NK cells and increased proportions of memory CD8 T cells, whereas IL-15 KO mice lack NK cells and have decreased CD8 T cells [16,17]. The ability of IL-15 to promote both NK cell and CD8 T cell responses has led to interest in IL-15 as a cancer immunotherapy. Most *in vivo* studies investigating the effects of IL-15 have used subcutaneous engrafted or lung metastasis cancer models. For example, several studies found that IL-15 TG mice were resistant to engrafted tumor formation [18,19]. IL-15 has been administered by several routes and use of each of these methods has impaired tumor growth or metastasis [20-25]. The protection observed was either NK cell and/or CD8 T cell dependent [18-20,22]. While many treatment strategies have been successful in engrafted and metastatic models, it is unknown if this will translate into a spontaneous epithelial cancer model where tumors initiate and grow alongside an intact tolerized immune system.

In this study, we crossed IL-15 KO and IL-15 TG mice with a spontaneous breast cancer model (MT) to create IL-15 KO/MT and IL-15 TG/MT mice. MT mice express the polyoma MT antigen under the mouse mammary tumor virus long terminal repeat [26]. In MT mice, multifocal adenocarcinomas form and these frequently metastasize to the lung [26]. The MT model on a C57BL/6 background is a good model of human breast cancer as tumor formation is sequential and goes from focal hyperplasia to mammary intraepithelial neoplasms to carcinoma *in situ* and ends with multiple invasive tumors [27,28]. IL-15 KO/MT, MT and IL-15 TG/MT were followed for tumor formation and endpoint. We characterized the immune environment both systemically and intra-tumorally and determined the relative contribution of NK and CD8 T cells to the protection we observed in IL-15 TG/MT mice. Lastly, we confirmed that when human NK cells were exposed to a similar cytokine environment as was observed in IL-15 TG/MT tumors, they were capable of killing human breast tumor cells.

## Methods

### Animal models

Mice were bred and maintained in the McMaster Central Animal Facility in “clean” rooms with a 12 hour day/night schedule and standard temperature controls. Procedures were approved by the McMaster Animal Research Ethics Board and comply with the guidelines set out by the CCAC. MMTV-MT mice (Dr. Gendler, Mayo Clinic, AZ) were crossed to IL-15 KO (Taconic, Germantown, NY) and IL-15 TG mice (Dr. Caligiuri, Ohio State University, OH) to generate IL-15 KO/MT and IL-15 TG/MT mice (C57BL/6 background). C57BL/6 control mice were purchased from Charles River (Quebec, Canada).

### Tumors

In the subcutaneous model, a MT cell line, established from a spontaneous MMTV-MT tumor (Mayo Clinic, Arizona), was injected (1 × 10^5^) subcutaneously. Mice were monitored 3 times per week for tumor formation/endpoint. In the spontaneous model, mice were palpated weekly for tumor formation and endpoint (tumors >10 × 10 mm). To examine metastasis, lungs from each group of mice were harvested at 120 days of age, perfused with 2% paraformaldehyde, embedded and sectioned 2 times 100 μM apart. Haematoxylin and eosin (H&E) stained sections were scored as positive or negative for the presence of tumor cells.

### Histology/immunohistochemistry

Tumors were excised from multiple mice per group and embedded in Tissue-Tek® OCT (Sakura) or fixed in 2% paraformaldehyde. Fixed sections were stained with H&E (n > 10 per group). Immunohistochemistry was performed on OCT sections for CD8α (PharMingen- #550281; 1:50) and CD4 (PharMingen- #550278; 1:50) using a Rat on Mouse Kit (Biocare Medical- #RT517H). Colour was developed using an AEC Chromogen substrate solution (Sigma). For quantitation, 5 random fields of view per section were counted in 5 mice per group (blinded). A TUNEL assay (ApopTag In Situ Apoptosis Detection Kit- Millipore) was also performed as per manufacturer’s instructions on 3 size matched tumors per group.

### Flow cytometry

To generate single cell suspensions, tumors were excised, digested (3 mg/ml Collagenase A, 0.025 mg/ml DNase I (Roche) - 45 min., 37°C, with shaking), and filtered sequentially (70 μm, 40 μM). Spleens were also collected and a single cell suspension was created by squishing. Red blood cells were removed with ACK lysis buffer. Cells were incubated with anti-mouse CD16/32 (eBioscience- #14-0161-86) (1 in 100, 15 min, 4°C) and then stained for markers including: NK1.1, CD69, NKG2D, NKp46, CD8, CD4, CD3, CD44, PD-1, CD27, CD62L and IFNγ/Perforin (intracellular flow cytometry using BD-cytofix/cytoperm) (eBiosciences/BD Biosciences). For flow cytometry analysis of tumors, the first gate was drawn on singlets followed by CD45+ cells (leukocytes) to exclude tumor cells. Fluorescence minus one (FMOs) controls were performed for all experiments. Samples were run on the BD LSRII or CANTO flow cytometer and FlowJo (Tree Star, Ashland, OR) was used for analysis.

### T cell stimulation

Cells were isolated from spleens and tumors as described above and CD8 T cells were isolated using a CD8 T cell selection kit (Stem cell- #18753). Purity was assessed by flow cytometric analysis and was >80% for tumors and >90% for spleens. 96 well plates were coated overnight with purified 1 μg/ml anti-CD3 (eBioscience- #16-0031-82) and 5 μg/ml anti-CD28 (eBioscience- #16-0281-82) antibodies at 4°C. The next day, plates were washed and 5 × 10^5^ CD8 T cells/well were added. Supernatants were collected 48 hours later. In the case of intracellular flow cytometry, the same process was followed, but after 12 hours, GolgiStop (BD Biosciences) was added for 10 hours prior to flow staining.

### ELISA/Cytokine analysis

IFNγ (DY485), TNFα (DY410E) and IL-12 p40 (DY499) ELISAs were performed using Murine DuoSet Kits from R&D Systems (Minneapolis, MN) as per manufacturer’s instruction. For IL-18 levels, 3 tumors per group (size matched) were homogenized and pooled in an equal volume and sent to Rules Based Medicine (RBM, Austin, TX) for multi-analyte protein analysis (RodentMAP® version 2.0).

### NK/Tumor Cell Killing assay

#### Mouse

NK cells were isolated from the spleens of C57BL/6 mice (PE selection kit Stem cell- #18551 with NK1.1-PE, 2 μg/ml). The MT cell line was labelled with 5-6-carboxyfluorescein diacetate succinimidyl ester (CFSE, 5 μM) and incubated with the NK cells at various Effector:Target ratios (1:1, 5:1, 10:1) for 5 hours (MT cells alone = basal lysis). After 5 hours, 7-amino actinomycin D (7-AAD, BD Biosciences, 5 μl/tube) was added and flow cytometry was performed to quantify the percentage of CFSE and 7-AAD positive cells (dead MT cells). Specific lysis was calculated via the following formula:

$$ \%\ specific\ lysis = \frac{100 \times \left(\%\ sample\ lysis - \%\ basal\ lysis\right)}{100 - \%\ basal\ lysis} $$

#### Human

Lymphocytes were isolated from PBMCs with Ficoll-Paque Plus (StemCell- #07907) and NK cells were selected with a human NK cell enrichment kit (StemCell- #19055, >90% purity of CD56 + CD3- NK cells). The resultant NK cells were cultured for 16 hours in IL-2 (100 U/ml) or IL-12 (10ng/ml)/IL-15 (20ng/ml)/IL-18 (100 ng/ml)(Peprotech). Cells were washed 4X before being cultured with CFSE (5 μM) labelled MDA-231 (NCI-60 panel) cells at various E:T ratios (1:1, 5:1, 10:1) for 5 hours (MDA-231 cells alone = basal lysis). Cells were then stained with CD45-PE (BD Biosciences) antibody and immediately before flow cytometry, 7-AAD (BD, 5 μl/tube) was added to identify dead cells. FMO controls were included in each experiment. Flow cytometry analysis was performed to determine PE-CFSE + 7AAD+ cells and the above formula was applied.

### Antibody depletion

For the subcutaneous model, mice were given 2 injections, one day apart of anti-NK1.1 (PK136 mouse IgG2, hybridoma HB191; ATCC) (200 μg/dose) antibody intraperitoneally. Two days later, MT cells were injected subcutaneously (and the 3^rd^ dose of NK1.1 antibody was administered). NK1.1 depleting antibody was delivered every 3-4 days to maintain the depletion. In the spontaneous model, IL-15 TG/MT mice were given 2 doses of 200 μg anti-NK1.1 mouse IgG antibody or 100 μg of anti-CD8α mouse IgG (clone 2.43; ATCC) intraperitoneally one day apart starting at 4 weeks of age. Depletion was continued every 3-4 days for the NK1.1 and once per week for the CD8 antibody for the duration of the experiment. At endpoint, animals were sacrificed and spleens/tumors were examined for depletion (n ≥ 3). Tumors were fixed (2% paraformaldehyde) and H&E sections were prepared.

### Statistical analysis

Statistics were performed in GraphPad Prism. T tests or One-way ANOVAs (Bonferonni’s post test) were performed, depending on the number of groups to be compared and error bars represent standard error of the mean (SEM). Survival curves were analyzed with the Log Rank test (Mantel-Cox).

## Results

### Overexpression of IL-15 leads to resistance to subcutaneous MT tumor formation, while lack of IL-15 leads to faster tumor growth compared to control mice

**Figure 1:**
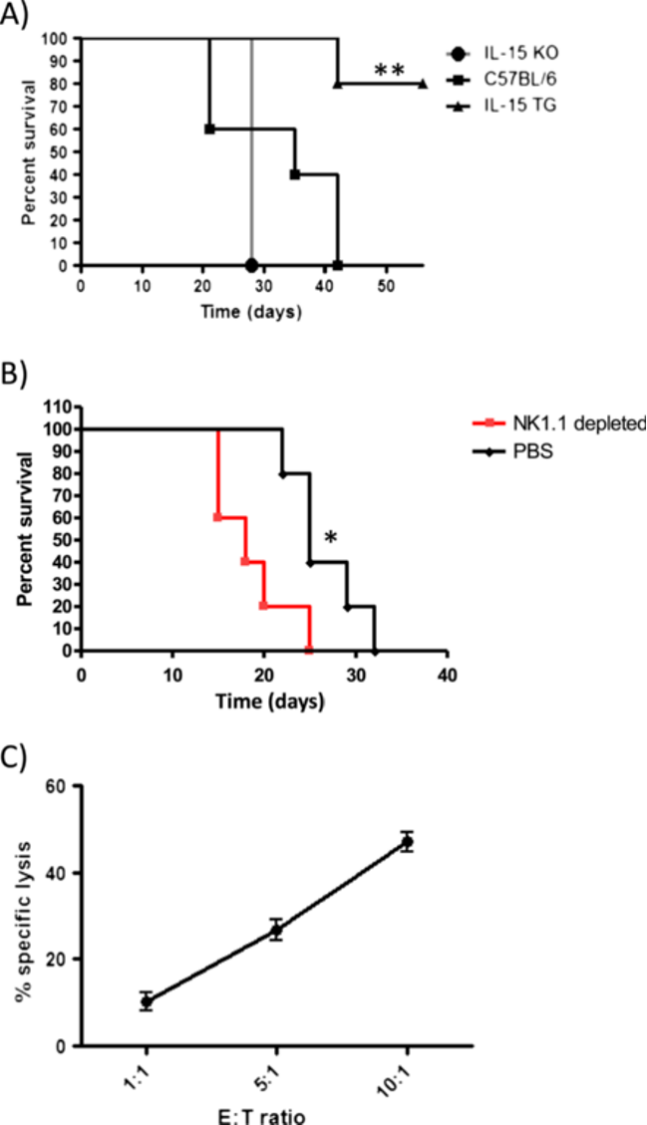
Subcutaneous breast tumor formation in IL-15 TG, IL-15 KO and C57BL/6 mice with MT cell line. **(A)** 1 × 10^6^ MT cells were injected subcutaneously into IL-15 KO, C57BL/6 and IL-15 TG mice (n = 5/group). IL-15 TG mice were resistant to tumor formation in comparison to either the IL-15 KO or C57BL/6 mice. **(B)** Depletion of NK cells in C57BL/6 mice via NK1.1 antibody led to decreased survival (n = 5/group). **(C)** NK cells isolated from the spleen of C57BL/6 mice (E-Effector) efficiently kill MT tumor cells (T-Target) in a killing assay (n = 3). *p < 0.05, **p < 0.01.

To determine if the presence or absence of IL-15 had an impact on the formation of subcutaneous breast tumors, C57BL/6, IL-15 KO and IL-15 TG mice were injected with 1 × 10^5^ MT cells subcutaneously and observed for endpoint. Most IL-15 TG mice were resistant to tumor formation (80% survival), whereas IL-15 KO mice proceeded to endpoint more quickly (n = 5/group) (Figure 1A). To determine if NK cells contributed to tumor protection, NK cells were depleted in control mice. NK cell depletion promoted tumor formation (data not shown) and decreased survival (Figure 1B). To further explore the role of NK cells, we tested whether NK cells were capable of killing MT tumor cells in a killing assay. At an E:T ratio of 10:1, we saw 47% specific lysis of MT cells by NK cells (Figure 1C). Therefore, NK cells can protect from this engrafted breast tumor model and are capable of killing MT cells specifically. We next sought to determine if this would extend to a spontaneous breast tumor model in which tumors form more similarly to those in humans [27].

### Overexpression of IL-15 leads to destruction of spontaneous mammary carcinoma

**Figure 2:**
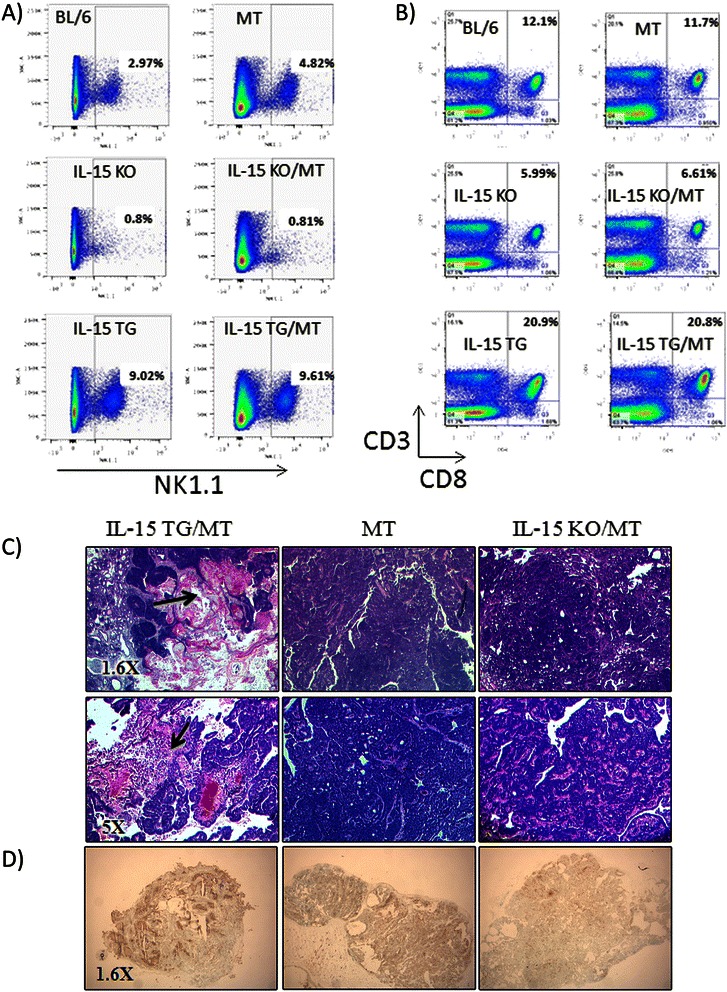
Impact of lack of IL-15 or overexpression of IL-15 on lymphocytes in the MT background and on tumor histology. **(A & B)** Spleens from 6-8 week old IL-15 KO, IL-15 KO/MT, BL/6, MT, IL-15 TG and IL-15 TG/MT were analyzed by flow cytometry for NK cells **(A)** and CD8 T cells **(B)** (representative of n = 3, IL-15 KO, BL/6 and IL-15 TG, n = 2 IL-15 KO/MT, MT and IL-15 TG/MT). Both IL-15 KO and IL-15 KO/MT mice have few NK cells and have reduced proportion of CD8 T cells. In contrast, IL-15 TG and IL-15 TG/MT mice both have an equal increase in NK cells and CD8 T cells proportions. **(C)** H&E staining of IL-15 KO/MT, MT and IL-15 TG/MT tumors. Representative images from n > 10 per group (1.6X and 5X). Arrows indicate areas of tumor destruction. **(D)** TUNEL stain (ApopTag In Situ Apoptosis Detection Kit- Millipore) on cryosections from representative IL-15 KO/MT, MT and IL-15 TG/MT tumors (n = 3/group) (Apoptosis = dark brown).

To establish the role of IL-15 in spontaneous breast tumor formation, MT mice were crossed to IL-15 KO and IL-15 TG mice to create IL-15 TG/MT and IL-15 KO/MT mice. To confirm that the IL-15 KO and IL-15 TG phenotype was maintained upon addition of the MT transgene, spleens from 6-8 week old mice were analyzed by flow cytometry. Both IL-15 KO and IL-15 KO/MT mice lack NK1.1 + CD3- NK cells and have decreased CD8 T cells (6% of total lymphocytes)(Figure 2A/B). In addition, IL-15 TG and IL-15 TG/MT mice both have similar amounts of NK cells (9% of total lymphocytes) and increased CD8 T cells (21% of total lymphocytes). In control C57BL/6 and MT mice there were 4-5% NK cells and 12% CD8 T cells.

To visualize the effect of IL-15 on tumor formation, tumors of all sizes were excised from IL-15 KO/MT, MT and IL-15 TG/MT mice and were sectioned for H&E staining (Figure 2C). IL-15 KO/MT tumors were solid, well vascularized tumors with very little cell death. In contrast, IL-15 TG/MT tumors were mainly necrotic with very few healthy tumor cells, had increased lymphocytic infiltration and TUNEL stain revealed cell death throughout (Figure 2D). The histological differences were observed in multiple tumors excised at various sizes (n > 10/group).

### IL-15 affects the speed of tumor formation, the progression to endpoint and metastasis in a spontaneous breast tumor model

**Figure 3:**
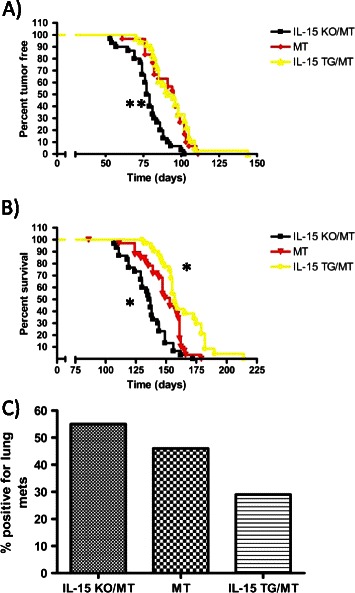
Percent tumor free **(A)**, survival curve **(B)** and percent with lung metastasis **(C)** for MT, IL-15 KO/MT and IL-15 TG/MT mice. Mice were palpated and tumors were measured once per week to determine date of first palpation or endpoint. **(A)** The median age of tumor palpation and number of mice per group was as follows; MT 95 (n = 30), IL-15 KO/MT 78 (n = 30) and IL-15 TG/MT 93 (n = 36). There is a statistical difference between the IL-15 KO/MT mice and the MT or IL-15 TG/MT mice (p < .0001). **(B)** The median age of endpoint and number of mice per group was as follows; MT 153 (n = 30), IL-15 KO/MT 136.5 (n = 30) and IL-15 TG/MT 157 (n = 28). In comparison to MT mice IL-15 TG/MT mice have increased survival (p = .0051), whereas IL-15 KO/MT mice have decreased survival (p = .002). **(C)** Percentage of mice with lung metastasis at 120 days of age (MT n = 13, IL-15 KO/MT n = 11 and IL-15 TG/MT n = 14). *p < 0.05, **p < 0.01.

The extensive histological differences between IL-15 TG/MT and IL-15 KO/MT mice indicated that IL-15 may play a role in tumor formation and progression to endpoint. In MT mice, multifocal mammary adenocarcinomas developed in 100% of mice at a median of 95 days and the median age of endpoint was 153 days (Figure 3A/B). IL-15 KO/MT tumors formed faster (median = 78 days) and progressed to endpoint more quickly than either MT or IL-15 TG/MT tumors (respectively, p < 0.0001, p < 0.01) (Figure 3A/B). IL-15 TG/MT mice had increased survival when compared to MT mice (p < 0.01) (Figure 3B).

In the MT mouse model, tumors frequently metastasize to the lung [26]. To examine metastasis in the absence or overexpression of IL-15, mice from each group were analyzed for lung tumor metastasis at 120 days of age. While not statistically significant, it is interesting to note that 29% of IL-15 TG/MT mice (n = 14), 46 % of MT mice (n = 13) and 55% of IL-15 KO/MT (n = 11) had detectable metastasis at this time point (Figure 3C).

### Tumors in IL-15 TG/MT mice have higher proportions of activated NK cells

**Figure 4:**
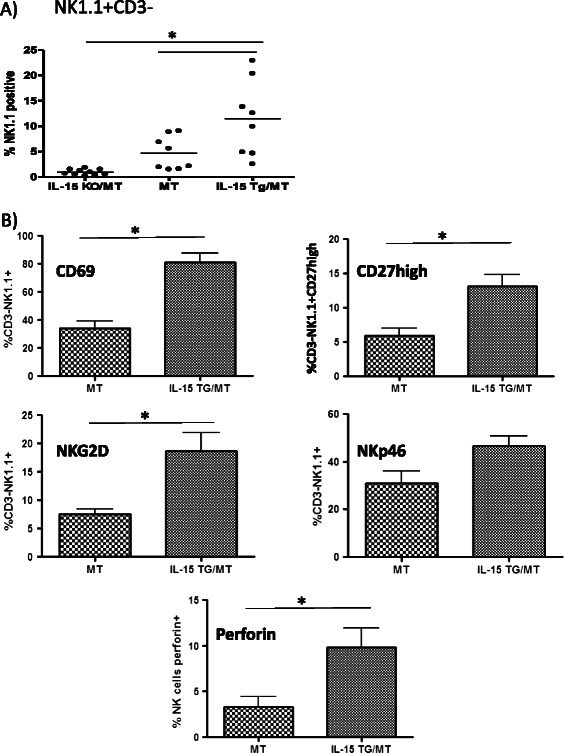
A comparison of NK cell phenotype in IL-15 TG/MT and MT tumors. Tumors from MT, IL-15 KO/MT and IL-15 TG/MT mice were digested and stained for flow cytometry (only one tumor taken from each mouse). **(A)** Of CD45+ cells in the tumor, there was a higher proportion of NK1.1+ cells in the IL-15 TG/MT tumors than in the MT or IL-15 KO/MT tumors (n = 10- IL-15 KO/MT, 8- MT or IL-15 TG/MT). **(B)** In the IL-15 TG/MT tumors, the majority of the NK cells possess the early activation marker CD69. In addition, a higher percentage of NK cells in IL-15 TG/MT tumors possess CD27, a marker of highly cytotoxic NK cells. Lastly, a higher percent of the NK cells in IL-15 TG/MT tumors possess the activation markers NKp46 and NKG2D, as well as perforin (n = 3 to 9). *p < 0.05.

Since IL-15 is known to promote the differentiation, survival and activation of NK cells, we examined the status of NK cells within these tumors [13,14,17]. Tumors were excised, digested and stained for flow cytometry markers to determine the proportion as well as the activation of NK cells (Figure 4). To analyze leukocyte populations alone, sample analysis was conducted on CD45+ cells. The IL-15 TG/MT tumors had a much higher proportion of NK1.1 + CD3- cells than MT tumors (p < 0.01) (Figure 4A). It has been shown that tumors possess or secrete several factors that can decrease NK cell activation [2,9,29]. We found that 80% of NK cells within the IL-15 TG/MT tumors possessed the early activation marker CD69, whereas <40% of MT NK cells were CD69+ (Figure 4B). In IL-15 TG/MT tumors, more NK cells expressed perforin, a key molecule indicative of cytotoxicity potential. In addition, a higher percentage of NK cells in IL-15 TG/MT tumors were in the CD27^high^ subgroup, which indicates an increased cytotoxicity and lower threshold of activation (Figure 4B) [30]. Lastly, the NK cells within IL-15 TG/MT tumors had higher levels of the activating receptors NKp46 and NKG2D (Figure 4B).

### IL-15 TG/MT tumors possess increased functional CD8 T cells

**Figure 5:**
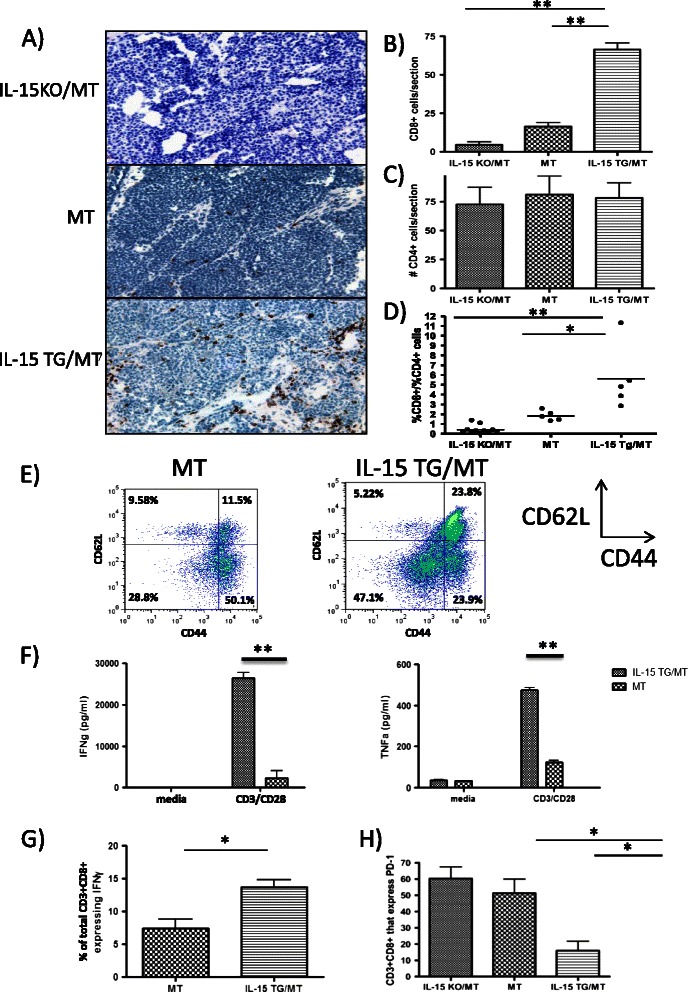
CD8 T cell phenotype in IL-15 TG/MT, IL-15 KO/MT and MT tumors. **(A)** CD8 T cell immunohistochemistry on representative IL-15 KO/MT, MT and IL-15 TG/MT tumors. Levels of CD8 T cells were highest in IL-15 TG/MT tumors and lowest in IL-15 KO/MT mice (brown staining = CD8+, 20X) **(B)** Quantitation of CD8 T cells in tumors (n = 5 mice/group) **(C)** Quantitation of CD4 T cells in tumors assessed by immunohistochemistry (n = 5 mice/group) **(D)** Tumors from MT, IL-15 KO/MT and IL-15 TG/MT mice were digested and stained for flow cytometry. The CD8+/CD4+ T cell ratio is altered drastically in the presence or absence of IL-15 (n = 5 mice/group). **(E)** CD3 + CD8+ cells from tumors of IL-15 TG/MT mice have a higher percentage of CD62L + CD44+ central memory T cells (Representative of n = 3/group) (F/G) CD8 T cells were isolated by positive selection from tumors of mice and stimulated non-specifically. **(F)** After 48 hours, supernatant IFNγ/TNFα levels were highest in CD8 T cells from IL-15 TG/MT mice (n = 2/group) (Representative of 3 experiments). **(G)** In another set of experiments, after 12 hours of stimulation, Golgi Stop was added to determine via flow cytometry which cells were capable of producing IFNγ. A high proportion of IL-15 TG/MT CD8 + CD3+ cells produce IFNγ in comparison to MT CD8 T cells (n = 3/group). **(H)** Fewer CD8 + CD3+ T cells in IL-15 TG/MT mice express the exhaustion marker PD-1 (n = 3/group). *p < 0.05, p < 0.01.

IL-15 is capable of stimulating the production of anti-tumor memory CD8 T cells [15,31]. Therefore, we wanted to determine if we had altered the T cell environment within the tumors when we altered the IL-15 levels. CD8α immunohistochemistry on IL-15 KO/MT, MT and IL-15 TG/MT tumor sections revealed that there were higher numbers of CD8 T cells within IL-15 TG/MT tumors in comparison to either IL-15 KO/MT or MT tumors (p < 0.001 for both) (Figure 5A/B). In contrast, there were approximately equal numbers of CD4 T cells in all of the tumor groups (Figure 5C). Researchers have found that a high CD8/CD4 ratio is a positive prognostic factor in tumors [32]. Using flow cytometry, the CD8 to CD4 ratio was found to be much higher for IL-15 TG/MT (mean = 5.63) than IL-15 KO/MT tumors (mean = 0.43) (Figure 5D).

To phenotype CD8 T cells in IL-15 TG/MT mice, we examined CD44 and CD62L expression. CD8 T cells in the majority of IL-15 TG/MT tumors possessed a CD44 + CD62L^high^ central memory population (>2 times higher than CD8 T cells of MT mice) (Figure 5E). The presence of CD8 T cells in the tumor is generally a positive prognostic factor, but CD8 T cells within tumors have been reported to be exhausted, regulatory or non-functional [33-35]. To test this, CD8 T cells were isolated from the spleens or tumors of IL-15 KO/MT, MT and IL-15 TG/MT mice, and stimulated non-specifically for 48 hours. CD8 T cells from IL-15 TG/MT spleens and tumors both produced higher levels of IFNγ and TNFα than those from MT mice (Figure 5F, Additional file 1: Figure S1C). IL-15 KO/MT spleen or tumor CD8 T cells produced little to no IFNγ (data not shown). We further examined the CD8 T cells via flow cytometry to determine which CD8 T cell populations were producing IFNγ (Figure 5G, Additional file 1: Figure S1). We isolated CD8 T cells and performed non-specific stimulation in the presence of GolgiStop (BD) to prevent protein secretion. A higher percentage of CD8 T cells from IL-15 TG/MT tumors produced IFNγ and IL-15 TG/MT tumors had a higher proportion of CD8 T cells with central memory phenotype markers (CD44 + CD62L+)(Figure 5G, Additional file 1: Figure S1A). After selecting the CD8 T cells that were capable of producing IFNγ, we determined that these cells were all CD44+, and in the case of IL-15 TG/MT, many were also CD62L+ (Additional file 1: Figure S1B). Therefore, IL-15 TG/MT mice have higher numbers of polyfunctional and central memory CD8 T cells. To ascertain if the CD8 T cells were exhausted, we examined them for the marker programmed death (PD)-1. This marker is increased on exhausted CD8 T cells in a variety of models [35]. PD-1 was high on intra-tumoral IL-15 KO/MT and MT CD8 T cells, but low on IL-15 TG/MT CD8 T cells (Figure 5H).

### Tumors in IL-15 TG/MT mice contain significant numbers of CD3 + CD8 + NK1.1+ cells

Phenotyping the CD8 T cells from these mouse strains revealed a large proportion of IL-15 TG/MT tumor CD8 T cells that also expressed NK1.1 (Additional file 1: Figure S2A). These NK1.1 + CD8 T cells were absent in IL-15 KO/MT tumors and much lower in MT tumors (Additional file 1: Figure S2A). To determine if these cells produced high levels of IFNγ, we isolated NK1.1+ cells from IL-15 TG/MT tumors and performed non-specific stimulation (CD3/28) in the presence of protein secretion inhibitors. 11-13% of CD3 + CD8+ NK1.1+ T cells produced IFNγ, a similar percent to that seen for total CD8 T cells that produced IFNγ (Additional file 1: Figure S2B, 5G). Therefore, in our model, these cells were capable of producing IFNγ, but were not the only CD8 T cells doing so.

### Cells expressing NK1.1 are responsible for tumor destruction in IL-15 TG/MT mice

**Figure 6:**
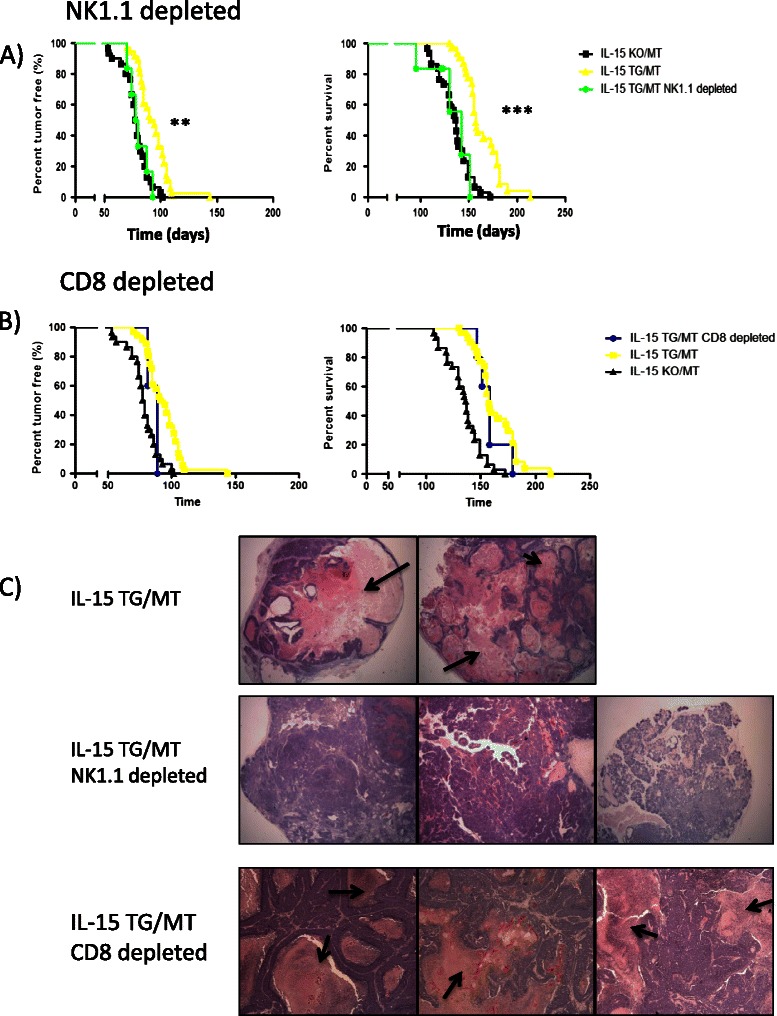
The effect of NK1.1 and CD8α depletion on tumor formation. **(A-B)** IL-15 TG/MT mice were depleted with anti-NK1.1 (n = 6) **(A)** or anti-CD8α (n = 5) **(B)** antibody long term starting at 4 weeks of age. **(A)** In comparison to the tumor formation and survival curves for IL-15 TG/MT mice (n = 36 for percent tumor free, n = 28 for survival), NK1.1 depleted IL-15 TG/MT mouse tumors formed and progressed to endpoint more quickly. IL-15 TG/MT NK1.1 depleted mice were not different from IL-15 KO/MT tumor mice (n = 30). **(B)** In comparison to the tumor formation and survival curves for IL-15 TG/MT mice, CD8 depleted IL-15 TG/MT mouse tumors formed and progressed to endpoint at a similar rate. **(C)** Tumors that formed in IL-15 TG/MT NK1.1 depleted mice did not show the extensive destruction seen in normal IL-15 TG/MT tumors and in IL-15 TG/MT CD8 depleted mice. Arrows indicate areas of tumor destruction. **p < 0.01, ***p < 0.001.

To determine which cell type(s) were responsible for anti-tumor responses in IL-15 TG/MT mice, we performed long term antibody depletion experiments with anti-NK1.1 or anti-CD8α antibody in IL-15 TG/MT mice. NK1.1 depletion efficiently depleted NK1.1+ cells within the spleen and tumor (Additional file 1: Figure S3A). IL-15 TG/MT mice that were depleted of NK1.1+ cells formed tumors faster and proceeded to endpoint more quickly than IL-15 TG/MT mice (p < 0.001, 0.0001, respectively) (Figure 6A). In fact, tumor formation closely followed what was observed in IL-15 KO/MT mice. In addition, histological analysis revealed that the tumor destruction in IL-15 TG/MT mice was absent when these NK1.1+ cells were absent (Figure 6C). The anti-CD8α antibody removed CD8 expressing cells from the spleen of IL-15 TG/MT mice, but only partially from the tumor (at least by 2/3 ie. 18% to 6%)(Additional file 1: Figure S3B). The majority of the CD3 + CD8+ T cells that were left in the depleted tumor were NK1.1+ (Additional file 1: Figure S3C). There were no statistically significant differences between the IL-15 TG/MT and the IL-15 TG/MT CD8 depleted mice (Figure 6B). Lastly, the tumors that formed in IL-15 TG/MT CD8 depleted mice had similar tumor destruction to that seen in normal IL-15 TG/MT mice (Figure 6C). Therefore, NK1.1+ cells play a major role in the tumor destruction and extension of survival in IL-15 TG/MT mice, whereas CD8 T cells, although activated phenotypically, play less of a role.

### Adoptive transfer of CD8 T cells from IL-15 TG/MT mice does not lead to protection from tumor challenge

To further examine the impact of CD8 T cells from IL-15 TG/MT mice on tumor formation, we performed CD8 T cell adoptive transfers. C57BL/6 recipient mice were treated with cyclophosphamide to induce lymphopenia. 24 hours later CD8 T cells were isolated from the spleens/tumors of MT, IL-15 TG/MT and IL-15 TG mice, labelled with CFSE (5 × 10^6^ spleen, 1 × 10^6^ tumor) and injected IV. 24 hours after transfer, mice were challenged with a sub-cutaneous dose of fresh primary MT tumors from which immune cells had been removed. Mice were followed for tumor formation and endpoint. 8 days after challenge, several mice were sacrificed to ensure that the adoptive transfer was successful (4-8% of total CD8 T cells were CFSE positive - data not shown). There were no statistically significant differences in tumor formation or endpoint between control mice that received no CD8 T cells and mice that received CD8 T cells from MT, IL-15 TG/MT or IL-15 TG spleens or IL-15 TG/MT tumors (Additional file 1: Figure S4).

### The effect of cytokines on the ability of human NK cells to kill a human breast cancer cell line

**Figure 7:**
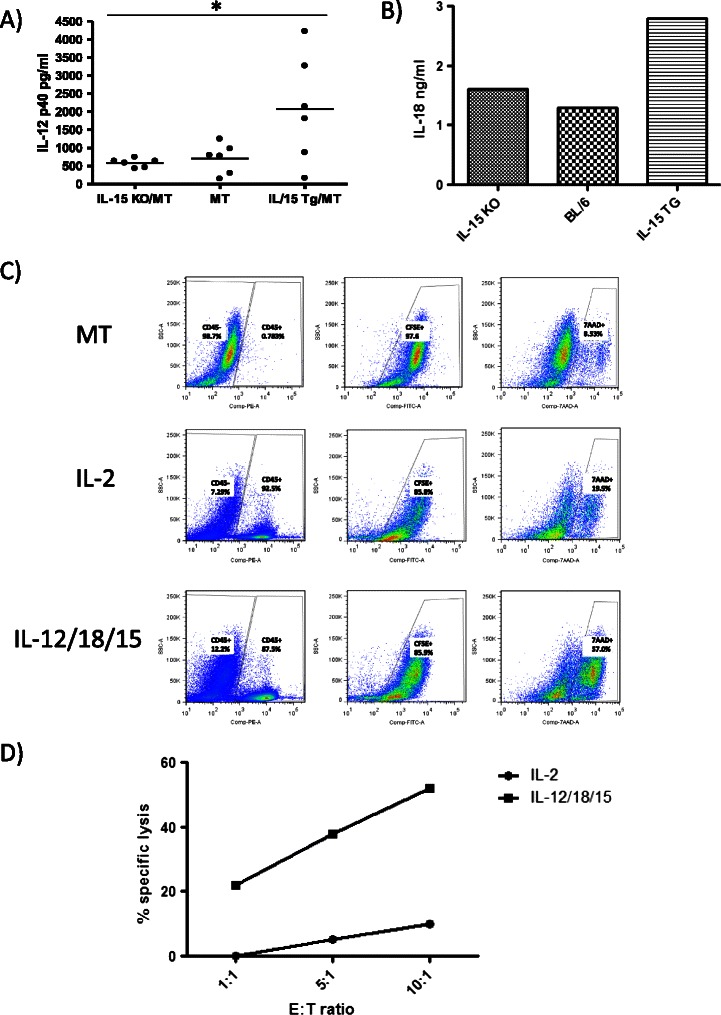
Cytokines in IL-15 TG/MT tumors and their effects on the ability of human NK cells to kill human breast cancer cells. **(A & B)** IL-12 and IL-18 are increased in IL-15 TG/MT tumors. **(A)** IL-12 ELISAs were performed on tumor homogenates from 6 size matched tumors per group. **(B)** 3 tumors from different mice in each tumor group were pooled (size matched between groups) and assayed for IL-18 cytokine levels via multianalyte protein analysis. **(C & D)** NK cells isolated from human PBMCs were cultured in IL-2 or IL-15/IL-12/IL-18 for 16 hours before being incubated with CFSE labelled MDA-231 cells for 5 hours in a killing assay. In the demonstration of the gating strategy in **C)** CD45+ NK cells were first removed from the analysis and then CFSE+ MT cells were selected. 7AAD was used to determine the percentage of the CFSE+ MT cells that were dead. Results at an E:T ratio of 1:10 are displayed (E:T = NKcell:MDA-231). MT alone is included to show spontaneous death levels which were used in the calculation of D. **(D)** Percent specific lysis of MDA-231 in the killing assay at various E:T ratios. Representative of 2 experiments. *p < 0.05.

IL-15 overexpression in MT breast tumors created an environment where several cytokines that affect NK cell activation and proliferation were altered. We examined the expression of these cytokines in IL-15 TG/MT tumors versus MT or IL-15 KO/MT tumors and found that in addition to IL-15, both IL-12 and IL-18 were increased within IL-15 TG/MT tumors (Figure 7A/B). To determine if exposure to these 3 cytokines simultaneously would affect the ability of NK cells to kill breast tumor cells, we isolated NK cells from human PBMCs and stimulated them in an IL-15/IL-12/IL-18 rich environment for 16 hours. We then co-incubated them with a CFSE labelled MDA-231 human breast cancer cell line at various E:T ratios for 5 hours to determine their killing potential. The gating strategy used can be seen in Figure 7C and included exclusion of CD45+ NK cells, selection of CFSE+ tumor cells and finally the percent of CFSE+ tumor cells that were dead (7AAD+). MT cells alone were included as the level of basal MT cell death. In contrast to NK cells cultured in IL-2 alone, NK cells cultured in IL-15/IL-12/IL-18 were highly cytotoxic toward this breast cancer cell line and at E:T ratios of 10:1 reached specific lysis levels of 52% (Figure 7C/D).

## Discussion

While IL-15 has been under investigation as a cancer immunotherapeutic for the last decade, investigation has focused on tumor models that do not closely mimic spontaneous tumor formation in humans. It is known that injecting tumor cell lines subcutaneously or intravenously is a useful, but rather artificial system in which it is easier to develop immune responses to the tumor. In addition, there is a lack of studies that have examined the impact of IL-15 on solid epithelial tumors such as breast cancer. To examine the role of IL-15 in a more relevant model we utilized an immunologically tolerant mouse model of spontaneous mammary tumor formation (MT) and examined tumor formation in the absence of IL-15 (IL-15 KO/MT) or with IL-15 overexpression (IL-15 TG/MT). Overall, IL-15 TG/MT mice had increased survival when compared to either MT or IL-15 KO/MT mice. In contrast IL-15 KO/MT mice had faster tumor formation and decreased survival when compared to MT or IL-15 TG/MT mice. These results are similar to the anti-tumor effects of IL-15 that have been observed in other engrafted and metastatic models of melanoma and colon cancer, but it is one of the first reports of this in a spontaneous model of breast cancer [18,19,21].

IL-15TG/MT mice formed tumors, but these tumors had a very different phenotype than IL-15 KO/MT or MT tumors. This included large areas of cell death, a higher proportion of NK cells as well as increased CD8 T cell infiltration. Increased CD8 T cells or NK cells within the tumor is a positive prognostic factor in many human and mouse tumor types [32,36,37]. In many cases though, NK cells within the tumor express inhibitory receptors instead of activating receptors and have low cytotoxicity [7,38]. To examine NK cell phenotype, we identified NK cells using flow cytometry as NK1.1 + CD3- cells. NK1.1 is commonly used as a marker for NK cells in C57BL/6 mice. It is a member of the NKRP1 receptor family and while it is thought to be an activating receptor its’ ligand is unknown [39-41]. The NK cells within IL-15 TG/MT tumors possessed higher levels of both activating receptors (NKG2D, NKp46) and other markers of activation (CD69). Recently, ligands for NKG2D and NKp46 were found to be expressed on human primary breast tumors and breast tumor cell lines [9]. In addition, blockade of either NKp46 or NKG2D decreased the ability of NK cells to kill breast tumor cells that expressed ligands to these receptors [9]. We also found that there was a higher percentage of CD27^high^ NK cells in IL-15 TG/MT tumors than in MT tumors. CD27 expression, in addition to CD11b expression, has been used to define mature mouse NK cells into subsets [30,42]. In progression from less mature to more mature: CD27^low^CD11b^low^ to CD27^high^CD11b^low^ to CD27^high^CD11b^high^ to CD27^low^CD11b^high^ [42]. Importantly, CD27^high^ NK cells were found to have a higher degree of effector function, including cytotoxicity and cytokine production [30]. More recently it was found that CD27 on human NK cells could also be used to define NK cell subsets [43,44]. In contrast to what has been found with mouse NK cells, human CD27+ NK cells have been associated with low cytotoxic activity and high ability to secrete cytokines [43,44]. Overall, NK cells found within IL-15 TG/MT tumors are likely more capable of killing breast tumor cells than those found in MT tumors.

We observed increased CD8 T cells within IL-15 TG/MT tumors, but CD8 T cells within the tumor are not always functional as they can be anergic and/or exhausted [33,35]. While IL-15 KO/MT tumor CD8 T cells had high levels of exhaustion markers (PD-1) and lacked IFNγ production, IL-15 TG/MT CD8 T cells had very low levels of PD-1 and produced large amounts of IFNγ. This is in contrast to another report that found that treatment with IL-15 in a metastatic model of colon carcinoma led to increased PD-1 expression on CD8 T cells in the spleen [45]. It is likely that the short term administration of IL-15 or the model system used accounted for the discrepancies in our observations. In addition, a higher proportion of CD8 T cells in the IL-15 TG/MT tumors were CD44+ and CD62L^high^, which are markers of central memory CD8 T cells. Central memory CD8 T cells are thought to be extremely effective in anti-tumor defence [31,46]. We also observed a high proportion of unique NK1.1+ CD8 T cells in IL-15 TG/MT tumors. This cell type has been previously identified as highly cytotoxic (high perforin/granzyme level) and able to produce large amounts of IFNγ [47,48]. In our model, while some of these cells produce IFNγ, they were not the major source. While we have not examined this here, it is interesting to speculate about whether these unique CD8 T cells developed in the tumor or migrated to the tumor from elsewhere. We do know that a low percentage of these cells can be found in IL-15 TG mice in other organs such as the spleen (data not shown), so they are not completely unique to the tumor environment. The ability of IL-15 to induce expression of other NK cell markers such as CD56 in human CD8 T cells has also been reported in a variety of models [49,50]. Thus, this effect does not appear to be limited to our mouse models or to one type of NK cell receptor.

In previous studies involving IL-15, protective effects were found to be NK cell or CD8 T cell dependent [18,19,22]. In IL-15 TG/MT mice, NK1.1 positive (includes NK cells, NKT cells, NK1.1+ CD8 T cells) but not CD8 positive cells were the most important cells for increased survival and tumor destruction. The CD8 depletion was substantial but not complete in the tumor and the majority of CD8 T cells that remained in the tumor were NK1.1+, indicating that this cell type was resistant to depletion via this method. It has previously been reported that these cells may be resistant to activation-induced cell death and this may contribute to the inefficient depletion [48]. Since CD3 + CD8 + NK1.1+ cells were removed by the NK1.1 depletion, we cannot rule out a role for this cell type in the tumor destruction of IL-15 TG/MT mice. The lack of contribution of CD8 T cells to increased survival was surprising due to the fact that they existed in such high numbers and were of the correct phenotype to fight cancer. To confirm this data, we performed a CD8 T cell adoptive transfer experiment. Despite the fact that CFSE labelled CD8 T cells were present in the spleen and tumor at endpoint (data not shown), there was no impact on survival from transfer of either IL-15 TG/MT, MT or IL-15 TG splenic CD8 T cells or IL-15 TG/MT tumor CD8 T cells after a subcutaneous primary MT tumor injection. It is possible that either this aggressive tumor formed too fast for the transferred CD8 T cells to have an impact or that the tumor rapidly lost MHC I expression to compensate for the presence of tumor specific CD8 T cells. Also, MT tumor formation has been found to be slightly different each time and different tumors may express different tumor antigens, some even lose expression of MT itself [51]. Thus, it is possible that despite taking MT tumor cells to inject from multiple mice and CD8 T cells from multiple mice and pooling them, they may still not have been specific for that tumor. In terms of MHC I loss, a similar phenomenon may be occurring in the spontaneous model. It is also possible that IL-15 overexpression may induce non-specific proliferation of CD8 T cells, not tumor specific responses [52]. This data indicates that overexpression of IL-15 can generate an anti-tumoral NK cell response that is effective at extending survival in the MT model.

Another promising finding revealed in this model was that overexpression of IL-15 appears to delay the formation of lung metastases. This observation, in a spontaneous model of breast tumor metastasis, strongly indicates that IL-15 has potential therapeutically to prevent metastasis. Previously, this has only been examined in injected models of metastasis. This may be very useful in a clinical setting in which metastasis is frequent and leads to significant increases in mortality.

## Conclusions

IL-15 is a promising new cancer therapeutic that is well tolerated in primate models [53]. It appears to be superior to IL-2 as it has lower toxicity, does not increase T regulatory cells and induces higher levels of NK cell and CD8 T cell effector responses [53,54]. Based on the success of IL-15 in animal models, the first clinical trials have begun (NCT01727076, NCT01021059, NCT01572593) in multiple tumor types including melanoma, renal cell carcinoma and non-small cell lung carcinoma patients. Recently, there has been renewed interest in NK cells as a target to activate in the fight against breast cancer. It has been shown that human breast cancer cells express activating ligands as well as death-inducing receptors- both of which NK cells use to correctly identify their target cells [9,55]. In fact, expression of NKG2D ligands in human breast cancer was associated with a significant beneficial outcome [56]. It has also been established that NK cells are capable of eradicating a solid epithelial cancer (fibrosarcoma) and that they may also be able to target breast cancer stem-cell like cells [4,5]. Here, we found that when human NK cells were exposed to a similar cytokine environment to that found in IL-15 overexpressed MT tumors, they were highly capable of killing a triple negative breast cancer cell line. Other studies have reported that NK cells grown in IL-15, IL-12 and IL-18 are thought to display long term effector functions and may be memory-like NK cells [57,58]. These studies, along with our data, lends credence to the idea that stimulating innate immune cells such as NK cells can be effective clinically against breast cancer primary tumors and metastasis.

## Additional file

Additional file 1: Figure S1. IFNγ production by CD8 T cells in the tumor and the spleen of IL-15 TG/MT and MT mice. **Figure S2.** IL-15 TG/MT tumors possess high levels of CD8+CD3+NK1.1+ T cells. **Figure S3.** Depletion efficiency in the spleen and tumor of IL-15 TG/MT mice when depleted with anti-NK1.1 (A) or anti-CD8α (B/C) antibody long term starting at 4 weeks of age (representative flow plots from n = 3 in each). **Figure S4.** Adoptive transfer of CD8 T cells from IL-15 TG/MT, MT or IL-15 TG mice does not protect from tumor formation.

## References

[1] Kiessling R, Klein E, Wigzell H (1975). “Natural” killer cells in the mouse. I. Cytotoxic cells with specificity for mouse Moloney leukemia cells. Specificity and distribution according to genotype. Eur J Immunol.

[2] Waldhauer I, Steinle A (2008). NK cells and cancer immunosurveillance. Oncogene.

[3] Vivier E, Tomasello E, Baratin M, Walzer T, Ugolini S (2008). Functions of natural killer cells. Nat Immunol.

[4] Liu RB, Engels B, Arina A, Schreiber K, Hyjek E, Schietinger A, Binder DC, Butz E, Krausz T, Rowley DA (2012). Densely granulated murine NK cells eradicate large solid tumors. Cancer Res.

[5] Li M, Knight DA, Smyth MJ, Stewart TJ (2012). Sensitivity of a novel model of mammary cancer stem cell-like cells to TNF-related death pathways. Cancer Immunol Immunother.

[6] Konjevic G, Jurisic V, Jovic V, Vuletic A, Mirjacic Martinovic K, Radenkovic S, Spuzic I (2012). Investigation of NK cell function and their modulation in different malignancies. Immunol Res.

[7] Mamessier E, Sylvain A, Thibult ML, Houvenaeghel G, Jacquemier J, Castellano R, Goncalves A, Andre P, Romagne F, Thibault G (2011). Human breast cancer cells enhance self tolerance by promoting evasion from NK cell antitumor immunity. J Clin Invest.

[8] Mamessier E, Bertucci F, Sabatier R, Birnbaum D, Olive D (2012). "Stealth" tumors: Breast cancer cells shun NK-cells anti-tumor immunity. Oncoimmunology.

[9] Mamessier E, Sylvain A, Bertucci F, Castellano R, Finetti P, Houvenaeghel G, Charaffe-Jaufret E, Birnbaum D, Moretta A, Olive D (2011). Human breast tumor cells induce self-tolerance mechanisms to avoid NKG2D-mediated and DNAM-mediated NK cell recognition. Cancer Res.

[10] Gillard-Bocquet M, Caer C, Cagnard N, Crozet L, Perez M, Fridman WH, Sautes-Fridman C, Cremer I (2013). Lung tumor microenvironment induces specific gene expression signature in intratumoral NK cells. Front Immunol.

[11] Bruno A, Focaccetti C, Pagani A, Imperatori AS, Spagnoletti M, Rotolo N, Cantelmo AR, Franzi F, Capella C, Ferlazzo G (2013). The proangiogenic phenotype of natural killer cells in patients with non-small cell lung cancer. Neoplasia.

[12] Konjevic G, Spuzic I (1993). Stage dependence of NK cell activity and its modulation by interleukin 2 in patients with breast cancer. Neoplasma.

[13] Ranson T, Vosshenrich CA, Corcuff E, Richard O, Muller W, Di Santo JP (2003). IL-15 is an essential mediator of peripheral NK-cell homeostasis. Blood.

[14] Cooper MA, Bush JE, Fehniger TA, VanDeusen JB, Waite RE, Liu Y, Aguila HL, Caligiuri MA (2002). In vivo evidence for a dependence on interleukin 15 for survival of natural killer cells. Blood.

[15] Judge AD, Zhang X, Fujii H, Surh CD, Sprent J (2002). Interleukin 15 controls both proliferation and survival of a subset of memory-phenotype CD8(+) T cells. J Exp Med.

[16] Fehniger TA, Suzuki K, Ponnappan A, VanDeusen JB, Cooper MA, Florea SM, Freud AG, Robinson ML, Durbin J, Caligiuri MA (2001). Fatal leukemia in interleukin 15 transgenic mice follows early expansions in natural killer and memory phenotype CD8+ T cells. J Exp Med.

[17] Kennedy MK, Glaccum M, Brown SN, Butz EA, Viney JL, Embers M, Matsuki N, Charrier K, Sedger L, Willis CR (2000). Reversible defects in natural killer and memory CD8 T cell lineages in interleukin 15-deficient mice. J Exp Med.

[18] Yajima T, Nishimura H, Wajjwalku W, Harada M, Kuwano H, Yoshikai Y (2002). Overexpression of interleukin-15 in vivo enhances antitumor activity against MHC class I-negative and -positive malignant melanoma through augmented NK activity and cytotoxic T-cell response. Int J Cancer.

[19] Kobayashi H, Dubois S, Sato N, Sabzevari H, Sakai Y, Waldmann TA, Tagaya Y (2005). Role of trans-cellular IL-15 presentation in the activation of NK cell-mediated killing, which leads to enhanced tumor immunosurveillance. Blood.

[20] Bessard A, Sole V, Bouchaud G, Quemener A, Jacques Y (2009). High antitumor activity of RLI, an interleukin-15 (IL-15)-IL-15 receptor alpha fusion protein, in metastatic melanoma and colorectal cancer. Mol Cancer Ther.

[21] Ugen KE, Kutzler MA, Marrero B, Westover J, Coppola D, Weiner DB, Heller R (2006). Regression of subcutaneous B16 melanoma tumors after intratumoral delivery of an IL-15-expressing plasmid followed by in vivo electroporation. Cancer Gene Ther.

[22] Epardaud M, Elpek KG, Rubinstein MP, Yonekura AR, Bellemare-Pelletier A, Bronson R, Hamerman JA, Goldrath AW, Turley SJ (2008). Interleukin-15/interleukin-15R alpha complexes promote destruction of established tumors by reviving tumor-resident CD8+ T cells. Cancer Res.

[23] Chang CM, Lo CH, Shih YM, Chen Y, Wu PY, Tsuneyama K, Roffler SR, Tao MH (2010). Treatment of Hepatocellular Carcinoma with Adeno-associated Virus Encoding Interleukin-15 Superagonist. Hum Gene Ther.

[24] Tang F, Zhao LT, Jiang Y, de Ba N, Cui LX, He W (2008). Activity of recombinant human interleukin-15 against tumor recurrence and metastasis in mice. Cell Mol Immunol.

[25] Dubois S, Patel HJ, Zhang M, Waldmann TA, Muller JR (2008). Preassociation of IL-15 with IL-15R alpha-IgG1-Fc enhances its activity on proliferation of NK and CD8+/CD44high T cells and its antitumor action. J Immunol.

[26] Guy CT, Cardiff RD, Muller WJ (1992). Induction of mammary tumors by expression of polyomavirus middle T oncogene: a transgenic mouse model for metastatic disease. Mol Cell Biol.

[27] Lin EY, Jones JG, Li P, Zhu L, Whitney KD, Muller WJ, Pollard JW (2003). Progression to malignancy in the polyoma middle T oncoprotein mouse breast cancer model provides a reliable model for human diseases. Am J Pathol.

[28] Xia J, Tanaka Y, Koido S, Liu C, Mukherjee P, Gendler SJ, Gong J (2003). Prevention of spontaneous breast carcinoma by prophylactic vaccination with dendritic/tumor fusion cells. J Immunol.

[29] Liu C, Yu S, Zinn K, Wang J, Zhang L, Jia Y (2006). Murine mammary carcinoma exosomes promote tumor growth by suppression of NK cell function. J Immunol.

[30] Hayakawa Y, Smyth MJ (2006). CD27 dissects mature NK cells into two subsets with distinct responsiveness and migratory capacity. J Immunol.

[31] Klebanoff CA, Finkelstein SE, Surman DR, Lichtman MK, Gattinoni L, Theoret MR, Grewal N, Spiess PJ, Antony PA, Palmer DC (2004). IL-15 enhances the in vivo antitumor activity of tumor-reactive CD8+ T cells. Proc Natl Acad Sci U S A.

[32] Nelson BH (2008). The impact of T-cell immunity on ovarian cancer outcomes. Immunol Rev.

[33] Srinivasan M, Frauwirth KA (2009). Peripheral tolerance in CD8+ T cells. Cytokine.

[34] Wang RF (2008). CD8+ regulatory T cells, their suppressive mechanisms, and regulation in cancer. Hum Immunol.

[35] Ahmadzadeh M, Johnson LA, Heemskerk B, Wunderlich JR, Dudley ME, White DE, Rosenberg SA (2009). Tumor antigen-specific CD8 T cells infiltrating the tumor express high levels of PD-1 and are functionally impaired. Blood.

[36] Street SE, Zerafa N, Iezzi M, Westwood JA, Stagg J, Musiani P, Smyth MJ (2007). Host perforin reduces tumor number but does not increase survival in oncogene-driven mammary adenocarcinoma. Cancer Res.

[37] Marrogi AJ, Munshi A, Merogi AJ, Ohadike Y, El-Habashi A, Marrogi OL, Freeman SM (1997). Study of tumor infiltrating lymphocytes and transforming growth factor-beta as prognostic factors in breast carcinoma. Int J Cancer.

[38] Carrega P, Morandi B, Costa R, Frumento G, Forte G, Altavilla G, Ratto GB, Mingari MC, Moretta L, Ferlazzo G (2008). Natural killer cells infiltrating human nonsmall-cell lung cancer are enriched in CD56 bright CD16(-) cells and display an impaired capability to kill tumor cells. Cancer.

[39] Vogler I, Steinle A (2011). Vis-a-vis in the NKC: genetically linked natural killer cell receptor/ligand pairs in the natural killer gene complex (NKC). J Innate Immun.

[40] Bartel Y, Bauer B, Steinle A (2013). Modulation of NK cell function by genetically coupled C-type lectin-like receptor/ligand pairs encoded in the human natural killer gene complex. Front Immunol.

[41] Sun JC, Lanier LL (2011). NK cell development, homeostasis and function: parallels with CD8(+) T cells. Nat Rev Immunol.

[42] Chiossone L, Chaix J, Fuseri N, Roth C, Vivier E, Walzer T (2009). Maturation of mouse NK cells is a 4-stage developmental program. Blood.

[43] Fu B, Tian Z, Wei H (2014). Subsets of human natural killer cells and their regulatory effects. Immunology.

[44] Vossen MT, Matmati M, Hertoghs KM, Baars PA, Gent MR, Leclercq G, Hamann J, Kuijpers TW, van Lier RA (2008). CD27 defines phenotypically and functionally different human NK cell subsets. J Immunol.

[45] Yu P, Steel JC, Zhang M, Morris JC, Waldmann TA (2010). Simultaneous blockade of multiple immune system inhibitory checkpoints enhances antitumor activity mediated by interleukin-15 in a murine metastatic colon carcinoma model. Clin Cancer Res.

[46] Klebanoff CA, Gattinoni L, Torabi-Parizi P, Kerstann K, Cardones AR, Finkelstein SE, Palmer DC, Antony PA, Hwang ST, Rosenberg SA (2005). Central memory self/tumor-reactive CD8+ T cells confer superior antitumor immunity compared with effector memory T cells. Proc Natl Acad Sci U S A.

[47] Assarsson E, Kambayashi T, Sandberg JK, Hong S, Taniguchi M, Van Kaer L, Ljunggren HG, Chambers BJ (2000). CD8+ T cells rapidly acquire NK1.1 and NK cell-associated molecules upon stimulation in vitro and in vivo. J Immunol.

[48] Ohta N, Hiroi T, Kweon MN, Kinoshita N, Jang MH, Mashimo T, Miyazaki J, Kiyono H (2002). IL-15-dependent activation-induced cell death-resistant Th1 type CD8 alpha beta + NK1.1+ T cells for the development of small intestinal inflammation. J Immunol.

[49] Correia MP, Costa AV, Uhrberg M, Cardoso EM, Arosa FA (2010). IL-15 induces CD8+ T cells to acquire functional NK receptors capable of modulating cytotoxicity and cytokine secretion. Immunobiology.

[50] Ohkawa T, Seki S, Dobashi H, Koike Y, Habu Y, Ami K, Hiraide H, Sekine I (2001). Systematic characterization of human CD8+ T cells with natural killer cell markers in comparison with natural killer cells and normal CD8+ T cells. Immunology.

[51] Maglione JE, McGoldrick ET, Young LJ, Namba R, Gregg JP, Liu L, Moghanaki D, Ellies LG, Borowsky AD, Cardiff RD (2004). Polyomavirus middle T-induced mammary intraepithelial neoplasia outgrowths: single origin, divergent evolution, and multiple outcomes. Mol Cancer Ther.

[52] Ramanathan S, Gagnon J, Ilangumaran S (2008). Antigen-nonspecific activation of CD8+ T lymphocytes by cytokines: relevance to immunity, autoimmunity, and cancer. Arch Immunol Ther Exp (Warsz).

[53] Berger C, Berger M, Hackman RC, Gough M, Elliott C, Jensen MC, Riddell SR (2009). Safety and immunologic effects of IL-15 administration in nonhuman primates. Blood.

[54] Mueller YM, Petrovas C, Bojczuk PM, Dimitriou ID, Beer B, Silvera P, Villinger F, Cairns JS, Gracely EJ, Lewis MG (2005). Interleukin-15 increases effector memory CD8+ t cells and NK Cells in simian immunodeficiency virus-infected macaques. J Virol.

[55] Kajitani K, Tanaka Y, Arihiro K, Kataoka T, Ohdan H (2012). Mechanistic analysis of the antitumor efficacy of human natural killer cells against breast cancer cells. Breast Cancer Res Treat.

[56] de Kruijf EM, Sajet A, van Nes JG, Putter H, Smit VT, Eagle RA, Jafferji I, Trowsdale J, Liefers GJ, van de Velde CJ (2012). NKG2D ligand tumor expression and association with clinical outcome in early breast cancer patients: an observational study. BMC Cancer.

[57] Cooper MA, Elliott JM, Keyel PA, Yang L, Carrero JA, Yokoyama WM (2009). Cytokine-induced memory-like natural killer cells. Proc Natl Acad Sci U S A.

[58] Ni J, Miller M, Stojanovic A, Garbi N, Cerwenka A (2012). Sustained effector function of IL-12/15/18-preactivated NK cells against established tumors. J Exp Med.

