# Supplementary material for: Overexpression of IL-15 promotes tumor destruction via NK1.1+ cells in a spontaneous breast cancer model
Source: BMC Cancer. 2015 Apr 16;15:293. doi: 10.1186/s12885-015-1264-3 (PMC4428091; doi:10.1186/s12885-015-1264-3)
Supplement: Additional file 1: Figure S1. — IFNγ production by CD8 T cells in the tumor and the spleen of IL-15 TG/MT and MT mice. Figure S2. IL-15 TG/MT tumors possess high levels of CD8+CD3+NK1.1+ T cells. Figure S3. Depletion efficiency in the spleen and tumor of IL-15 TG/MT mice when depleted with anti-NK1.1 (A) or anti-CD8α (B/C) antibody long term starting at 4 weeks of age (representative flow plots from n = 3 in each). Figure S4. Adoptive transfer of CD8 T cells from IL-15 TG/MT, MT or IL-15 TG mice does not protect from tumor formation. [file 12885_2015_1264_MOESM1_ESM.pdf]

S1

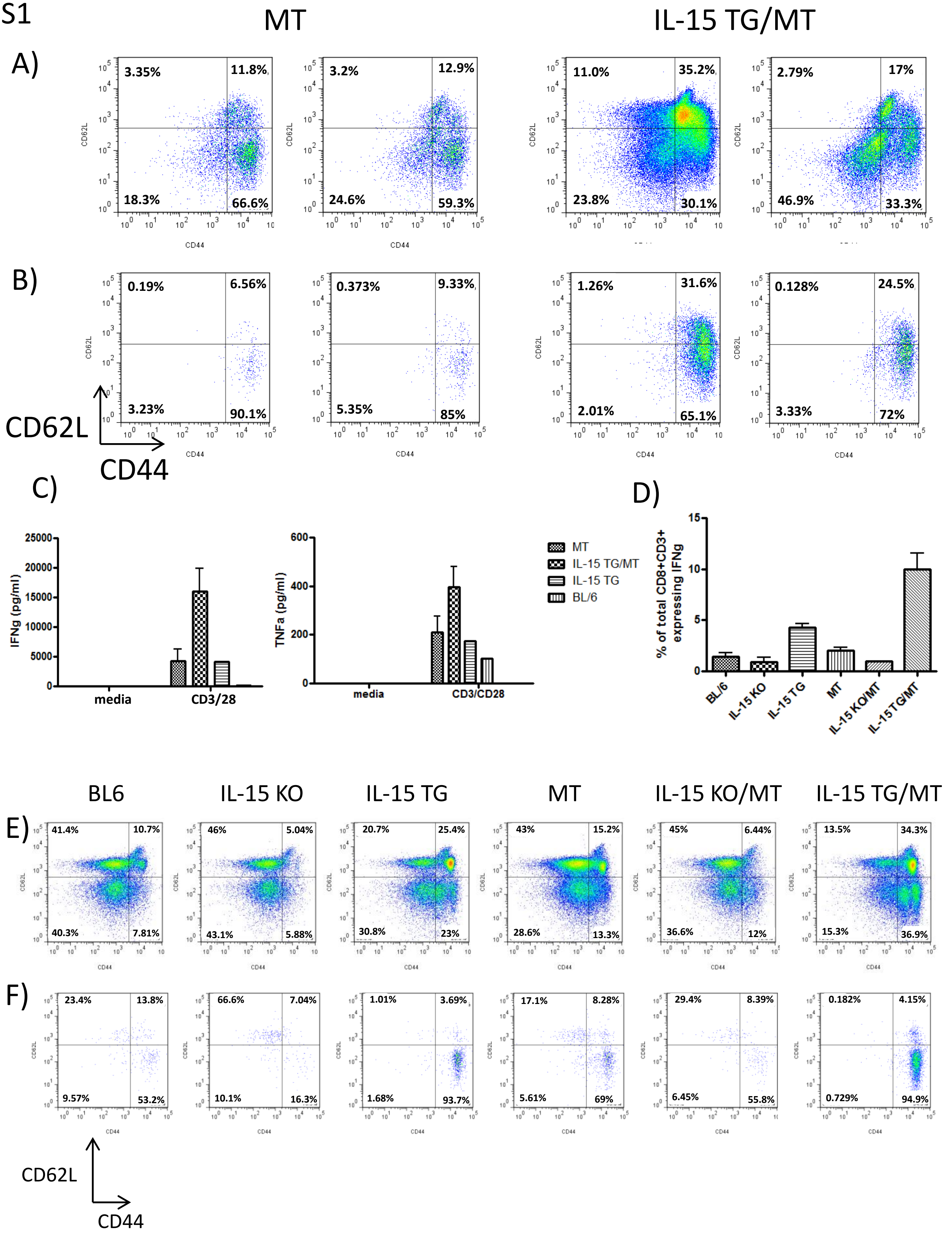
MT
IL-15 TG/MT
A)
3.35%
11.8%
18.3%
66.6%
3.2%
12.9%
24.6%
59.3%
11.0%
35.2%
23.8%
30.1%
2.79%
17%
46.9%
33.3%
B)
0.19%
6.56%
3.23%
90.1%
0.373%
9.33%
5.35%
85%
1.26%
31.6%
2.01%
65.1%
0.128%
24.5%
3.33%
72%
CD62L
CD44
C)
IFNg (pg/ml)
media
CD3/28
TNFa (pg/ml)
media
CD3/CD28
MT
IL-15 TG/MT
IL-15 TG
BL/6
D)
% of total CD8+CD3+ expressing IFNg
BL/6
IL-15 KO
IL-15 TG
MT
IL-15 KO/MT
IL-15 TG/MT
BL6
IL-15 KO
IL-15 TG
MT
IL-15 KO/MT
IL-15 TG/MT
E)
41.4%
10.7%
40.3%
7.81%
46%
5.04%
43.1%
5.88%
20.7%
25.4%
30.8%
23%
43%
15.2%
28.6%
13.3%
45%
6.44%
36.6%
12%
13.5%
34.3%
15.3%
36.9%
F)
23.4%
13.8%
9.57%
53.2%
66.6%
7.04%
10.1%
16.3%
1.01%
3.69%
1.68%
93.7%
17.1%
8.28%
5.61%
69%
29.4%
8.39%
6.45%
55.8%
0.182%
4.15%
0.729%
94.9%
CD62L
CD44

Supplemental Figure 1: IFNγ production by CD8 T cells in the tumor and the spleen of IL-15 TG/MT and MT mice. (A & B) CD8 T cells were isolated by positive selection from tumors of mice and stimulated non-specifically. (A) After stimulation, IL-15 TG/MT CD8 T cells had a higher proportion of CD44+CD62L+ (central memory) cells. (B) Of the CD8+CD3+ T cells, it was the CD44+, both CD62L positive or negative, cells that were capable of producing IFNγ. (n= 3/group) (C - F) CD8 T cells were isolated by positive selection from spleens of mice and stimulated non-specifically. (C) After 48 hours, supernatant IFNγ/TNFα levels were highest in IL-15 TG/MT spleen CD8 cultures (n=3 MT and IL-15 TG/MT, n=1 BL/6 and IL-15 TG-representative of 3 experiments). In another set of experiments, after 12 hours of stimulation, Golgi Stop was added to determine which cells were capable of producing IFNγ (D-F). (D) A higher proportion of IL-15 TG/MT splenic CD8+CD3+ cells produced IFNγ in comparison to MT CD8 T cells. (E) After stimulation, IL-15 TG/MT CD8 T cells had a higher proportion of CD44+ CD62L- (effector/effector memory) and CD44+CD62L+ (central memory) cells. (F) It is the CD44+ cells that are capable of producing IFNγ (D-F, n=3 MT and IL-15 TG/MT, n=2 BL/6, IL-15 KO, IL-15 TG, IL-15 KO/MT).

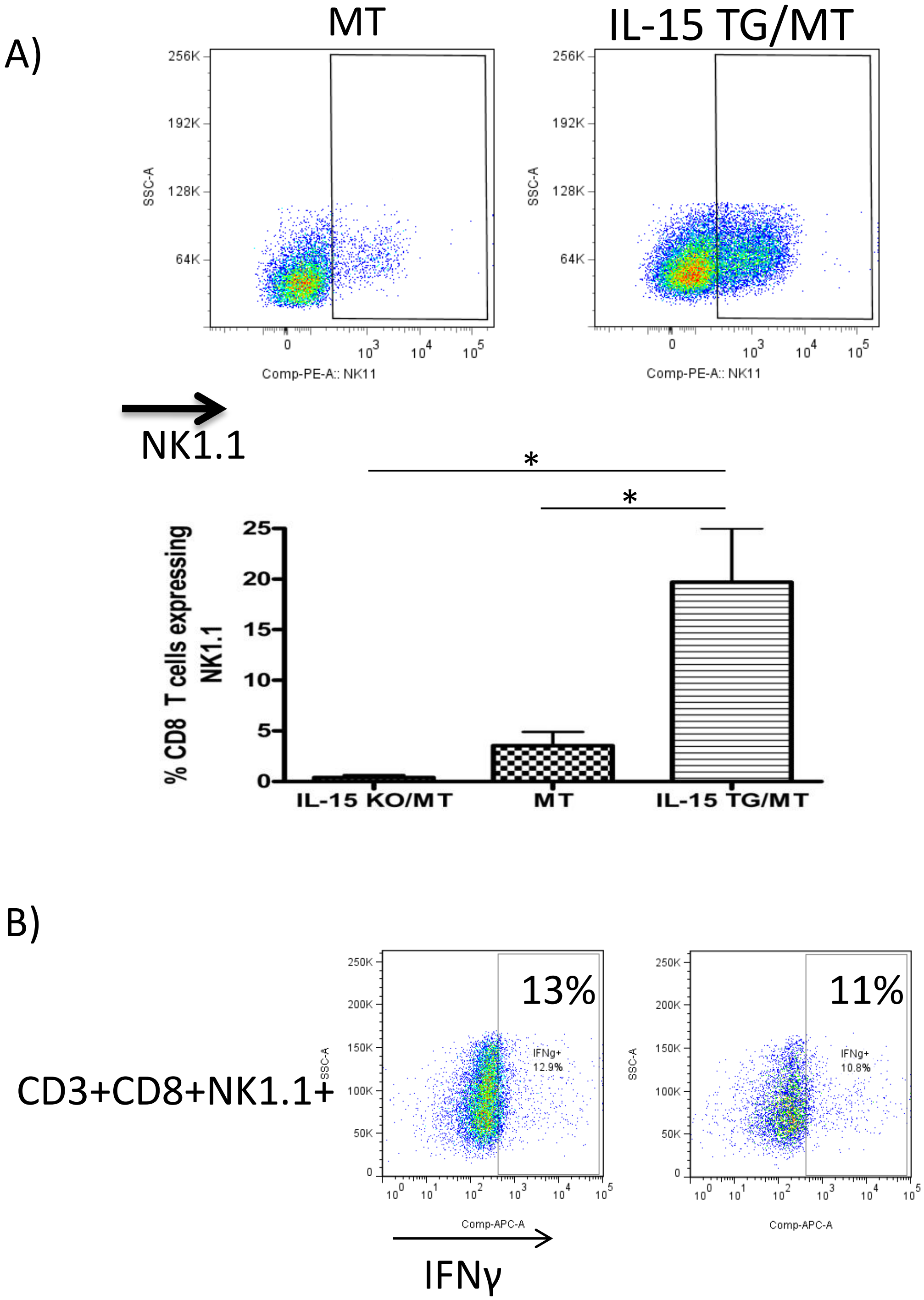

Supplemental Figure 2: IL-15 TG/MT tumors possess high levels of CD8+CD3+NK1.1+ T cells. (A) Tumors were digested and stained for CD45, CD8, CD3, and NK1.1 for flow cytometric analysis. IL-15 TG/MT tumors possessed high levels of unique CD8+CD3+NK1.1+ T cells (representative of 2 experiments, here n=2/group) (B) When CD8 T cells were isolated from IL-15 TG/MT tumors and stimulated non-specifically with CD3/CD28 antibodies in the presence of GolgiStop, 11-13% of these cells were found to produce IFNγ (n=2). *Statistically significant

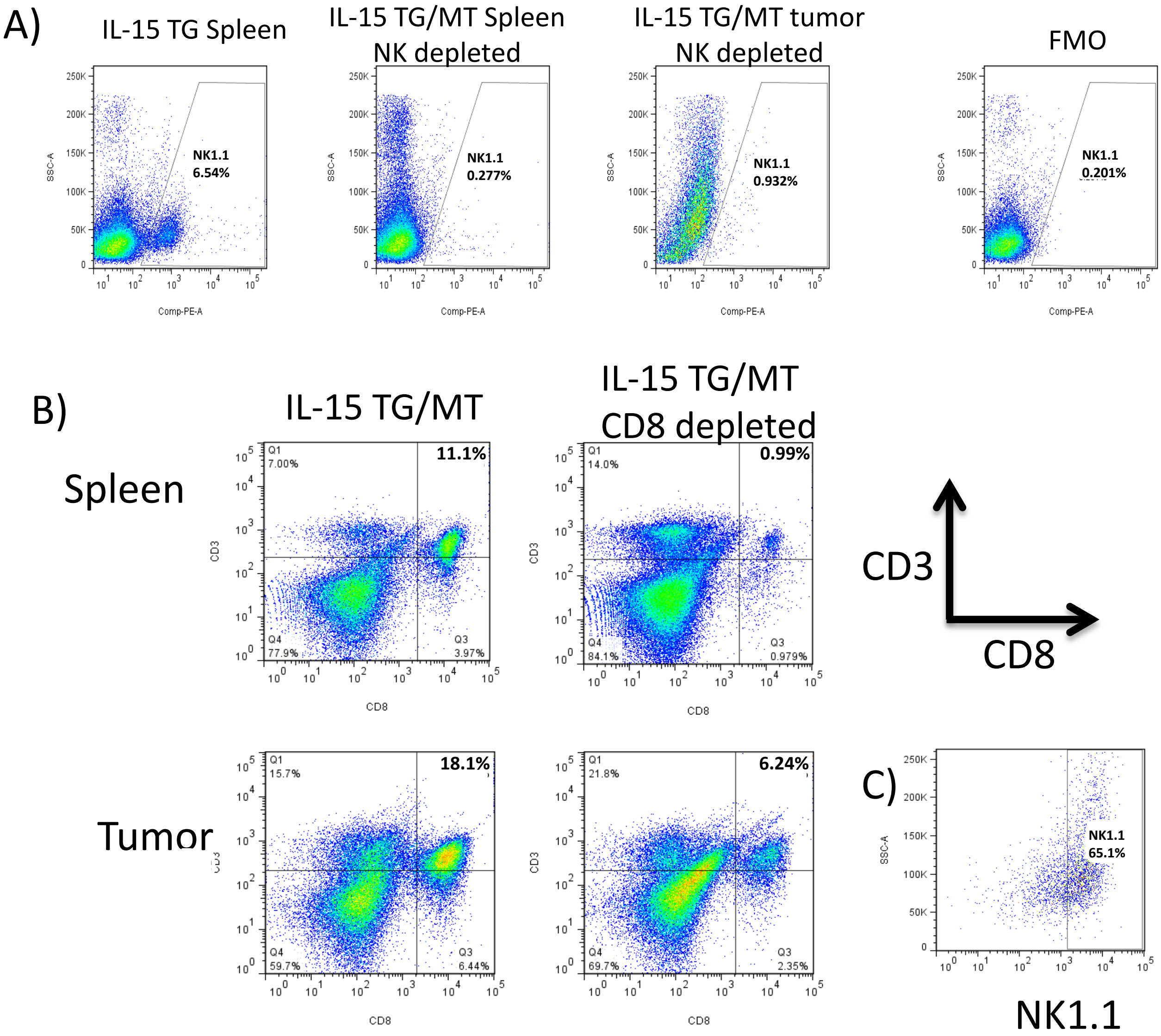

Supplemental Figure 3:IL-15 TG/MT mice were depleted with anti-NK1.1 (A) or anti-CD8α (B/C) antibody long term starting at 4 weeks of age (representative flow plots from n=3 in each). (A) Long term depletion efficiently removed NK1.1 positive cells from both the spleen and tumor (FMO= fluoresence minus one). (B) Long term depletion efficiently removed CD8 positive cells from the spleen, but not completely in the tumor. (C) Of the CD8+CD3+ cells that remained in the tumor, the majority were NK1.1+.

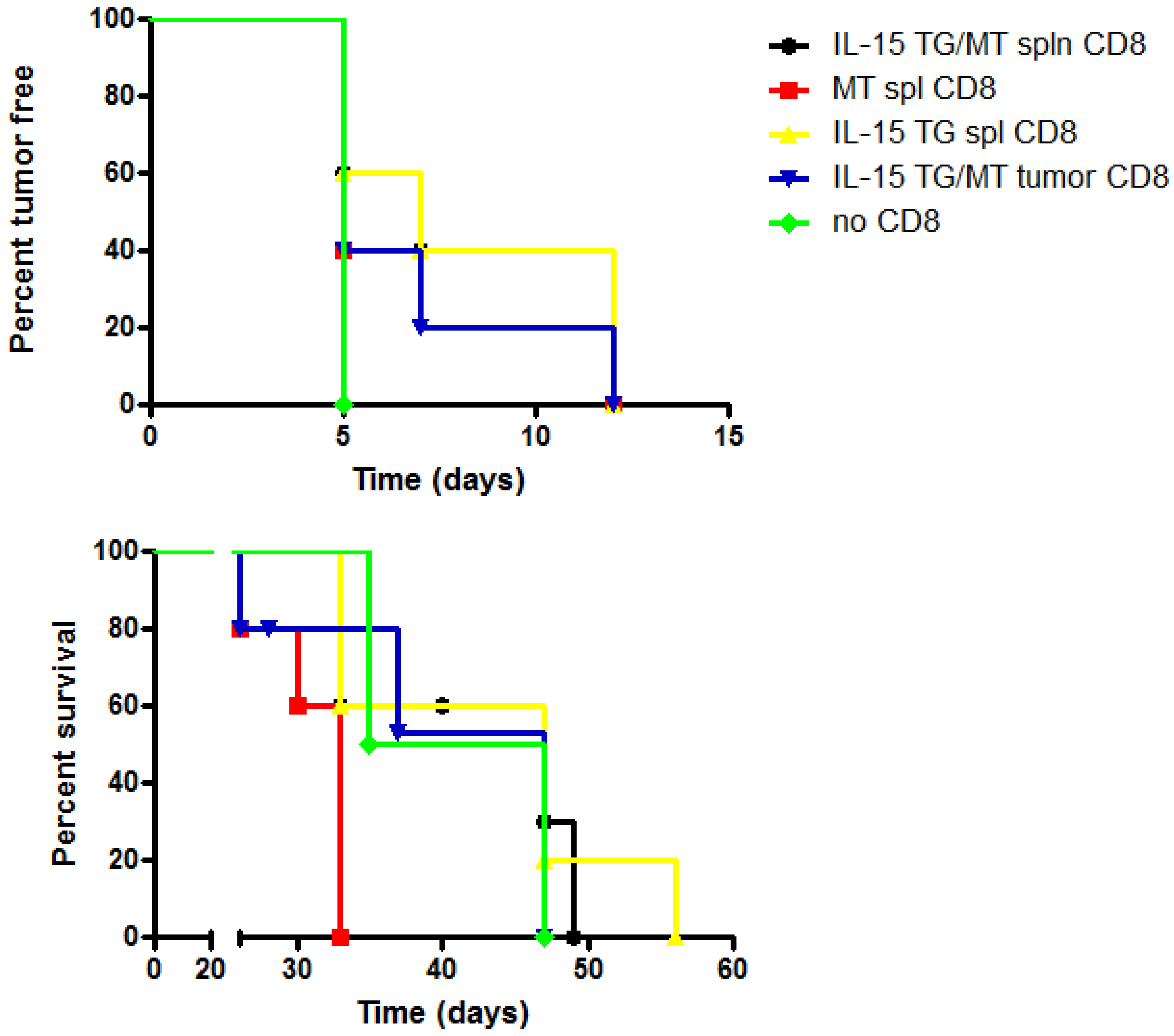

Supplemental Figure 4: Adoptive transfer of CD8 T cells from IL-15 TG/MT, MT or IL-15 TG does not protect from tumor formation. CD8 T cells were isolated from the spleens of IL-15 TG/MT, MT, IL-15 TG or tumors of IL-15 TG/MT mice. $5X10^6$ spleen CD8 T cells and $1X10^6$ tumor CD8 T cells were transferred into lymphopenic hosts. 24 hours later, mice were challenged with $0.5X10^6$ fresh primary MT cells subcutaneously. Resultant mice were followed for tumor formation (A) and endpoint (B). No statistically significant differences were found (n=5 for all groups except those receiving no CD8, n=2).
